# A Metabolic Signature to Monitor Endothelial Cell Differentiation, Activation, and Vascular Organization

**DOI:** 10.3390/biomedicines10092293

**Published:** 2022-09-15

**Authors:** Filipa Lopes-Coelho, Filipa Martins, Ana Hipólito, Sílvia V. Conde, Sofia A. Pereira, Luís G. Gonçalves, Jacinta Serpa

**Affiliations:** 1 iNOVA4Health, NOVA Medical School|Faculdade de Ciências Médicas, Universidade NOVA de Lisboa, Campo dos Mártires da Pátria, 130, 1169-056 Lisboa, Portugal; 2 Instituto Português de Oncologia de Lisboa Francisco Gentil (IPOLFG), Rua Prof Lima Basto, 1099-023 Lisboa, Portugal; 3 Instituto de Tecnologia Química e Tecnológica (ITQB) António Xavier da Universidade Nova de Lisboa, Av. da República, 2780-157 Oeiras, Portugal

**Keywords:** endothelial cells, monocytes, differentiation, sprouting, metabolic profile, metabolic signature, biomarkers

## Abstract

The formation of new blood vessels is an important step in the morphogenesis and organization of tissues and organs; hence, the success of regenerative medicine procedures is highly dependent on angiogenesis control. Despite the biotechnological advances, tissue engineering is still a challenge. Regarding vascular network formation, the regulators are well known, yet the identification of markers is pivotal in order to improve the monitoring of the differentiation and proliferation of endothelial cells, as well as the establishment of a vascular network supporting tissue viability for an efficacious implantation. The metabolic profile accompanies the physiological stages of cells involved in angiogenesis, being a fruitful hub of biomarkers, whose levels can be easily retrieved. Through NMR spectroscopy, we identified branched amino acids, acetate, and formate as central biomarkers of monocyte-to-endothelial-cell differentiation and endothelial cell proliferation. This study reinforces the successful differentiation process of monocytes into endothelial cells, allowing self-to-self transplantation of patient-derived vascular networks, which is an important step in tissue engineering, since monocytes are easily isolated and autologous transplantation reduces the immune rejection events.

## 1. Introduction

The formation of new vessels is a fundamental process for several biological phenomena, such as the various stages of embryonic development, regeneration processes, and some types of disease, cancer being the most widely studied [1,2,3,4,5,6,7]. An initial vascular network is based on the appearance of vessels with the simplest structure, the capillaries. These vessels basically have a monolayer of endothelium (*tunica intima*) lying on a basement membrane that may have associated pericytes (myofibroblasts), whose function is to contract and facilitate the movement of blood in small-caliber vessels [1].

The understanding of the molecular mechanisms regulating and ensuring the strategic points of activation of new vessel formation is of paramount importance. Hence, the processes of differentiation of endothelial cells, such as from monocytes that were recently described as ensuing endothelial progenitor cells [8], as well as the activation of *bona fide* endothelial cells in new vessel formation processes from pre-existing vessels [9,10], may provide an opportunity for clinical intervention.

Despite technological advances, regenerative medicine still faces several challenges [11]. One of them is the reproduction of tissue in the laboratory that can be implanted in humans to repair damage inflicted on tissues that are essential for the maintenance of the individual’s quality of life, such as musculoskeletal injuries [9,12,13] and injuries due to burns [14]. In fact, therapeutic angiogenesis has been explored to promote tissue revascularization in the course of bone injury treatment [13], but much more studies are needed. Some studies have been dedicated to the stimulation of angiogenesis in damaged tissues [15] in order to improve the healing potential, for instance, with the implantation of encapsulated cells expressing VEGF as an *in loco* attempt to activate angiogenesis [16] and a *quasi*-natural vascular network formation. As mentioned, the in-lab development of tissues to be used in humans is still a technical challenge, and the establishment of a functional vascular network supporting tissue architecture and growth is a pivotal step. Therefore, the in vitro differentiation of endothelial cells is essential, and if this process can be performed using the patient’s own cells, such as monocytes, it will contribute to a greater success of the procedure, as it reduces the risk of immune rejection [17]. Likewise, ensuring the formation of a network of vessels in vitro that keeps the tissue viable for grafts, in situations of regeneration and wound healing, configures an advance in the clinical management.

In all these processes, the molecular control is necessary, which makes the identification of markers crucial for monitoring the establishment of viable vessels and by ensuring the cellular and tissue conditions for transplantation. Therefore, these markers can be a powerful tool in controlling the quality, development staging, and maturation of tissue to be used in regenerative processes, contributing to the optimization of procedures and improving the treatment of patients.

**Figure 1:**
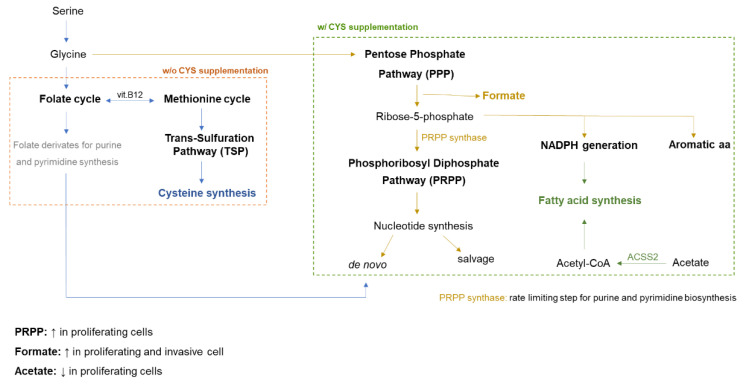
**Cysteine supplementation may be involved in the promotion of cell proliferation in monocytes, abrogating differentiation.** In environments with increased cysteine bioavailability, the glycine flux will be deviated to the pentose phosphate pathway (PPP), increasing the levels of ribose-5-phosphate (R5P) that contributes to nucleotide synthesis through the phosphoribosyl diphosphate pathway (PRPP). Since the nucleotide synthesis (*de novo* or savage) is dependent on ribose-5-phosphate for the PRPP, the shuttle from the folate/methionine cycle to the PPP will favor nucleotide demands essentially for cell proliferation. Although folate byproducts are needed for *de novo* nucleotide synthesis, PRPP is the rate-limiting reaction. In theory, proliferating cells will favor the PPP instead of the folate/methionine cycle to sustain the PRPP through R5P. Moreover, the more active PPP leads to more formate production (metabolite increased in cysteine-supplemented monocytes), and NADPH generated from the PPP will supply fatty acid synthesis and acetyl-CoA derived from acetate (metabolite decreases in cysteine-supplemented monocytes).

The metabolic profiling of cells is dynamic and varies according to their physiological status [6], and recently, we described that oxidative stress and compounds that interfere with the antioxidant status of monocytes and endothelial cells are important players in endothelial cell differentiation and activation [8,18]. Hence, monitoring the levels of organic compounds produced by cells during differentiation, activation, and proliferation upon exposure to different conditions is the perfect setting to identify markers. Nuclear magnetic resonance (NMR) spectroscopy has revealed to be a reliable and easy-to-use technique for the metabolome definition of biological samples [19]. Therefore, we proposed to analyze, through NMR spectroscopy, cell extracts and cell culture media in order to find a signature allowing the identification of different physiological statuses of cells pivotal for the formation of new blood vessels, as monocytes and endothelial cells. Metabolic stressful conditions acting on angiogenesis, such as hypoxia-mimicked conditions and oxidative stress, were tested, as well as the role of cysteine as a pivotal organic compound, presenting a core role in metabolism (Figure 1), together with its role as an antioxidant. In addition, the metabolic profiles resulting from the activation of monocyte-to-endothelial-cell differentiation by disulfiram [8] and the inhibition of endothelial cells by propranolol [18] were investigated.

## 2. Material and Methods

### 2.1. Monocyte Isolation and Culture

Monocytes were isolated from peripheral blood (PB) collected under consent donation of healthy donors from Serviço de Imuno-Hemoterapia at Instituto Português de Oncologia de Lisboa Francisco Gentil (IPOLFG) (IPOLFG-Ethical Committee UIC-1137). PB mononuclear cells (PBMCs) from blood samples were separated using Histopaque-1077 (10771, Sigma-Aldrich; St. Louis, MI, USA), followed by magnetic monocyte isolation using Monocyte Isolation Kit II (130-091-153, MACS Technology—Miltenyi Biotec; Bergisch Gladbach, Germany), according to the manufacturers’ protocols. Monocytes were cultured in plates coated with 0.2% of gelatin (G-1890, Sigma-Aldrich) or with Matrigel (354230, Corning; Sigma-Aldrich; St. Louis, MI, USA), and maintained in a colony-forming unit (CFU) medium (130-091-277, MACS Technology—Miltenyi Biotec; Bergisch Gladbach, Germany) or endothelial basal medium 2 (EBM-2; CC-3156, Lonza; Basel, Switzerland) plus EGM-2 SingleQuots^TM^ Supplements (CC-4176, Lonza; Basel, Switzerland). Media were supplemented with 2% fetal bovine serum (FBS; CC4101A, Lonza; Basel, Switzerland), 50 ng/mL vascular endothelial growth factor (VEGF; V7259, Sigma-Aldrich; St. Louis, MI, USA), and 10 U/mL heparin (H3149, Sigma-Aldrich; St. Louis, MI, USA). Cells were maintained at 37 °C in a humidified atmosphere and 5% CO_2_. Hydrogen peroxide (15 μM, H_2_O_2_; 1.07210.0250, Merck KGaA; Darmstadt, Germany) was used as a ROS generator; cysteine (0.4 mM, CYS; 7048-04-6, Merck KGaA; Darmstadt, Germany) was used as an antioxidant, since it is a glutathione precursor [20,21] and a H_2_S donor [22,23]; disulfiram (2 μM; 86720, Fluka; Charlotte, NC, USA) was used as an inhibitor of ALDH (aldehyde dehydrogenase, a marker for endothelial progenitor cell stemness) [24] that works as an activator of monocyte-to-endothelial-cell differentiation [8]; and propranolol (100 μM; P8688; Merck KGaA) was used as an angiogenesis inhibitor [25].

### 2.2. Endothelial Cell Culture

Human umbilical vein endothelial cells (HUVECs: CRL-1730, ATCC; Manassas, VA, USA) and endothelial cells isolated from the sprouted aortic rings were cultured in endothelial cell growth basal medium 2 (EBM-2: CC-3156, Lonza; Basel, Switzerland) supplemented with EGM-2 SingleQuots Supplements (CC-4176, Lonza; Basel, Switzerland) and maintained at 37 °C in a humidified atmosphere of 5% CO_2_. Cells were used until passage 10 and were detached with 0.05% trypsin-EDTA 1X (25300-054, Invitrogen, Thermo Fisher Scientific, Waltham, MA, USA). For hypoxia-mimicked experimental conditions, cells were cultured in the presence and in the absence of 100 μM cobalt chloride (CoCl_2_; 232696, Merck KGaA; Darmstadt, Germany). Hydrogen peroxide (15 μM, H_2_O_2_; 1.07210.0250, Merck KGaA; Darmstadt, Germany) was used as a ROS generator, cysteine (0.4 mM, CYS; 7048-04-6, Merck KGaA; Darmstadt, Germany) was used as an antioxidant, and propranolol (100 μM, Prop; P8688; Merck KGaA; Darmstadt, Germany) was used as an angiogenesis inhibitor.

### 2.3. Tube Forming Assay Cell

Corning^®^ Matrigel^®^ Matrix (356234, Sigma-Aldrich; St. Louis, MI, USA) was plated onto 48-well plates at 37 °C for 30 min, and HUVECs were incubated with calcein (2 μg/mL; C1430, Invitrogen, Thermo Fisher Scientific, Waltham, MA, USA), a fluorescent cell permeable dye, for 30 min at 37 °C and 5% CO_2_. After that, trypsinized HUVECs (3 × 10^4^ cells/well) were harvested, suspended, and seeded into Matrigel and then incubated at 37 °C and 5% CO_2_ for 6 h. Representative images of vessel-like structure formation were acquired in an Olympus IX53 Inverted Microscope.

### 2.4. Rat Aortic Ring Sprouting Assay

Aortas (thoracic and abdominal segments) were dissected from male Wistar rats (10 weeks old) and cleaned to remove external tissue (ethical committee NOVA Medical School Ref. 75/2019/CEFCM). After removing all extraneous fat, fibrotic tissue, and vasa vasorum structures, the aorta was segmented into rings with approximately 1 mm length. The rings were transferred to a Petri dish and incubated overnight in an FBS-free culture medium at 37 °C, 5% CO_2_. On the next day, the rings were embedded in Corning^®^ Matrigel^®^ Matrix in a 24-well plate with an EBM-2 culture medium and exposed to the experimental conditions: 15 µM H_2_O_2_, 0.4 mM CYS, 200 μM CoCl_2_, or 100 μM propranolol. The medium was refreshed every 3–4 d (days), the sprouts being visible at 7–13 d. Representative images were acquired on an Olympus IX53 Inverted Microscope, and the branch point (intersections between ECs) number *per* area was counted using ImageJ (imagej.nih.gov/ij/, accessed on 1 February 2022). The density of vessel-like structure formation (branch point number/μm^2^) was calculated as proxy of vascular density.

### 2.5. Nuclear Magnetic Resonance (NMR)

Monocytes and HUVECs were harvested with 1× PBS (washed twice), scraped, and centrifuged at 155× *g* for 10 min. Aortic rings were collected from Matrigel and frosted. After defrosting, cold methanol and water were added, and the rings were mechanically disrupted with a cell homogenizer in ice, followed by vortex.

The cells from the sprouted aortic rings were harvested from Matrigel in an ice-cold environment. After liquefaction of Matrigel, the cell suspension was diluted in a culture medium and centrifuged at 155× *g* for 10 min. After culture conditions, cell extracts were performed.

The methanol and chloroform extraction was used to separate organic and aqueous phases. After cold methanol mixture (4 mL methanol/1 g weight pellet), 2 volumes of water were added, mixed, and incubated for 5 min on ice. Chloroform (1 volume) was added to the sample and mixed. Then, 1 volume of water was added, and samples were incubated for 10 min on ice, following centrifugation at 210× *g* for 15 min at 4 °C. Aqueous (upper) and organic (lower) phases were collected. The aqueous phase was dried on a Speed Vac Plus Scllon and then dissolved in KPi buffer (50 mM, pH 7.4) in deuterated water (D_2_O) with 4% (*v*/*v*) sodium azide (NaN_3_) and 0.097 mM of 3-(trimethylsilyl)propionic-2,2,3,3-d4, which was used as a chemical shift and concentration reference. Culture supernatants were also diluted in this solution at a 1:10 ratio. ^1^H spectra (noesypr1d) were obtained in a magnetic field of 500 MHz in Ultrashied^TM^ 500 Plus (Bruker) equipped with a TCI-Z probe and 800 MHz in Ultrashied^TM^ 800 Plus equipped with a TCI H&F/C/N cryoprobe (Bruker) at 25 °C. Compound identification was performed by resorting to the Human Metabolome Database (HMDB) (http://www.hmdb.ca/; accessed on 1 February 2022) and ChemomxNMRsuite 8.12, which was used for metabolite quantification.

### 2.6. Statistical Analysis

All data were analyzed using Student’s *t*-test (unpaired) or two-way ANOVA in a GraphPad Prism v8 software (www.graphpad.com/; accessed on 1 February 2022). The assays were performed with at least 3 biological replicates *per* condition, and the differences were determined to be statistically significant at *p*-value < 0.05. Multivariate analysis of data was performed using SIMCA^®^ (SIMCA 13.0.3 software; Umetrics, Umea, Sweden; www.sartorius.com/; accessed on 1 February 2022). Enrichment analysis was performed on MetaboAnalyst 5.0 [26] using metabolite concentrations as inputs.

## 3. Results

### 3.1. A Metabolic Signature Follows the Monocyte-to-Endothelial-Cell Differentiation

Since oxidative stress, within certain limits, is an activator of endothelial cells and angiogenesis [27,28,29], we explored the metabolic profile of monocyte differentiation into endothelial cells upon oxidative stress and the role of cysteine in metabolic rescue. Additionally, the impact of the activation of monocyte-to-endothelial-cell differentiation by disulfiram [8] and the inhibition of endothelial cells by propranolol [18] were analyzed.

**Figure 2:**
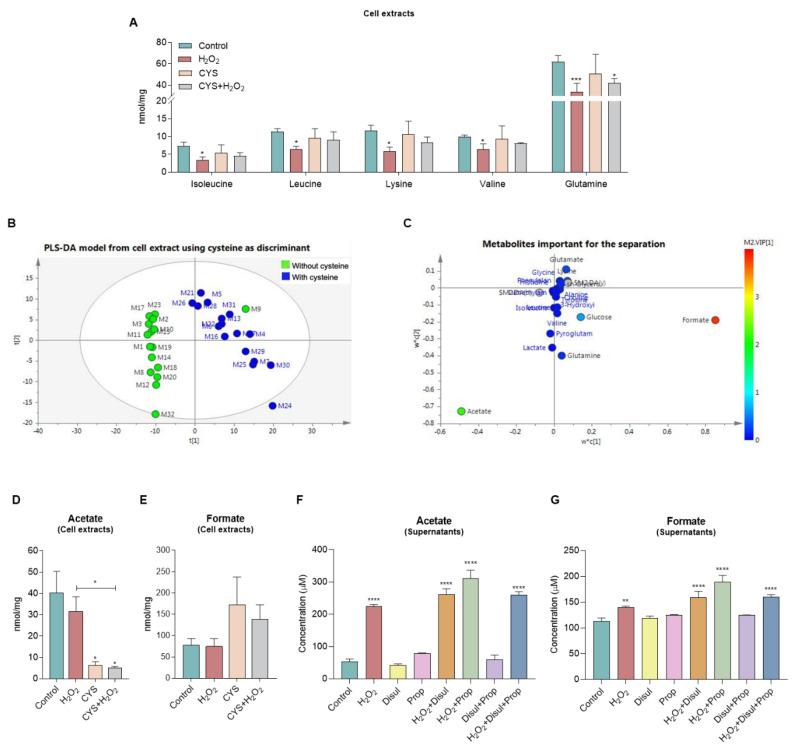
**Monocytes exposed to H_2_O_2_ decrease branched chain amino acids (BCAAs), valine, and glutamine catabolism, while cysteine (CYS) promotes an increase in formate levels and a decrease in acetate.** Monocytes were isolated and stimulated by hydrogen peroxide (15 μM, H_2_O_2_; ROS) during 30 min in the presence or absence of cysteine (0.4 mM), disulfiram (2 μM), or propranolol (100 μM). Disulfiram and propranolol incubation was performed for 30 min. Basal culture condition (control) and cysteine exposed cells were also analyzed. Cells were collected, cell extracts were performed, and cell culture media (supernatants) were collected for nuclear magnetic resonance (NMR) spectroscopy analysis. (**A**) Metabolite concentration of BCAAs (isoleucine, leucine, and lysine), valine, and glutamine of monocytes exposed to H_2_O_2_ and/or cysteine. (**B**) Multivariate analysis of monocytes cultured under H_2_O_2_ and/or cysteine—PLS-DA model from cell extracts using cysteine as discriminant. (**C**) Metabolites important for the separation of metabolic profiles. PLS-DA plots of first (*x* axis) and second (*y* axis) components. The score values determined the position of the different samples on the PLS-DA score plot, and the weight values the importance of the metabolites for the construction of the PLS-DA model. t1-PLS-DA score value of first component, t2-PLS-DA score value of second component, w*c1-PLS-DA X-weights and Y-weights values in the first component, w*c2-PLS-DA X-weights and Y-weights values in the first component. (**D**) Acetate levels upon H_2_O_2_ and/or cysteine from monocytes’ cellular extracts. (**E**) Formate levels upon H_2_O_2_ and/or cysteine from monocytes’ cellular extracts. (**F**) Impact of disulfiram and propranolol in acetate levels from monocytes’ supernatant. (**G**) Impact of disulfiram and propranolol in formate levels from monocytes’ supernatant. Results are represented as mean ± SD. * *p* < 0.05, ** *p* < 0.01, *** *p* < 0.001, **** *p* < 0.0001, unpaired *t*-test.

Through NMR spectroscopy, we observed that the differentiation pattern of monocytes upon H_2_O_2_ exposure drives a decrease in the intracellular concentration of branched chain amino acids (BCAAs) (isoleucine, leucine, and lysine), valine, and glutamine (Figure 2A), indicating that H_2_O_2_ promotes the consumption and catabolism of amino acids to support monocyte-to-endothelial-cell differentiation. Additionally, cysteine exposure rescues the low concentration of those metabolites, as noticed upon the simultaneous exposure to cysteine and H_2_O_2_ (Figure 2A). By using a multivariate analysis, it is observed that cysteine exposure promotes a metabolic shift (Figure 2B), having as main contributors the increased levels of formate and the decreased levels of acetate (Figure 2C).

The evaluation of the metabolic profile of monocyte extracts showed that acetate and formate levels were maintained upon ROS (H_2_O_2_) exposure, accompanying the differentiation process into endothelial cells, but after cysteine exposure, increased levels of formate and decreased levels of acetate were observed (Figure 2D,E), suggesting that cysteine blocks the differentiation process. In the monocyte cell culture media (supernatants), acetate and formate levels showed an increase due to ROS exposure; however, disulfiram and propranolol had no effect on acetate and formate levels (Figure 2F,G). All of these results suggest a metabolic remodeling commanding the proliferation of monocytes, as well as the monocyte-to-endothelial-cell differentiation process (Figure 1).

### 3.2. Upon Hypoxia, Endothelial Cells Present Metabolic Alteration Characteristic of Activation

**Figure 3:**
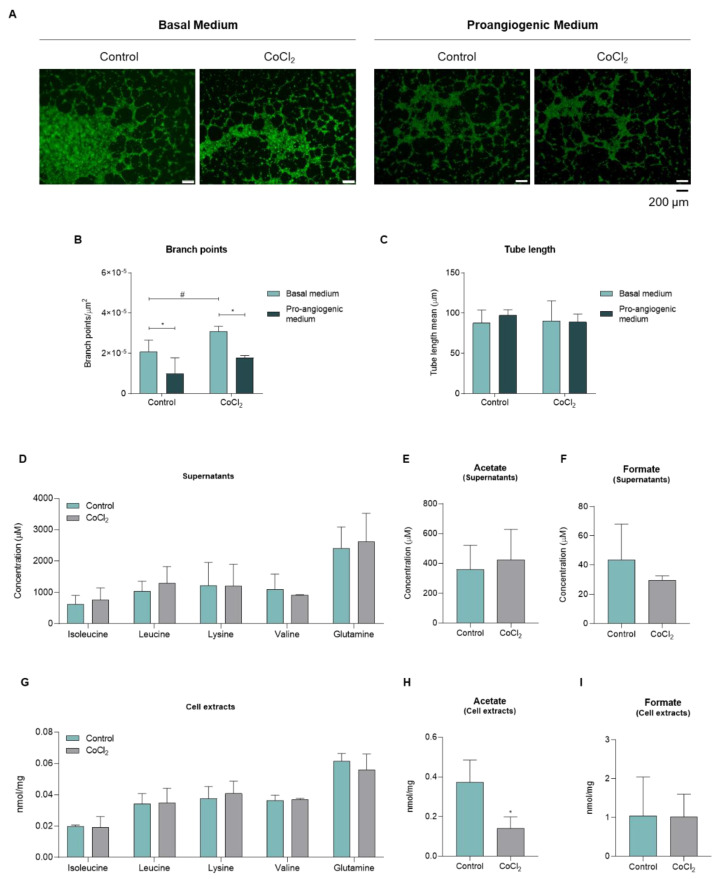
**Hypoxia potentiates the formation of vessel-like structures and decreases the extracellular acetate levels.** Human umbilical vein endothelial cells (HUVECs) were cultured in basal culture condition (control) and in hypoxia-mimicked condition (200 μM CoCl_2_). A tube forming assay was performed in Matrigel upon these culture conditions for 6 h. (**A**) Representative images (40×, scale: 200 μm) of vessel-like structures formed by calcein-labeled HUVECs. Cells were exposed to CoCl_2_-mimicked hypoxia and cultured in basal or proangiogenic medium. Quantification of the respective branch points (**B**) and tube length (**C**) of the newly formed vessel-like structures. HUVECs cultured in the same conditions were collected, and cell extracts were performed, and cell culture media (supernatants) were collected for nuclear magnetic resonance (NMR) spectroscopy analysis. (**D**) Concentration of amino acids detected in the supernatant of HUVECs treated with CoCl_2_. (**E**) Acetate levels in HUVECs’ supernatant. (**F**) Formate levels in HUVECs’ supernatant. (**G**) Concentration of amino acids detected in cellular extracts of HUVECs treated with CoCl_2_. (**H**) Acetate levels in HUVECs’ cellular extracts. (**I**) Formate levels in HUVECs’ cellular extracts. All data are represented as mean ± SD. (*) *p* < 0.05, two-way ANOVA; (#) *p* < 0.05, unpaired *t*-test.

In order to explore whether the metabolic pattern is representative of endothelial cell activation, the analysis of metabolic profiles was performed in HUVECs exposed to CoCl_2_-mimicked hypoxia, since hypoxia is a ROS-generating condition and is a relevant stimulus for angiogenesis. At first, it was confirmed that hypoxia induced tubelike structure formation and significantly increased the branch point density, although not affecting the tube length (Figure 3A–C). The BCAAs isoleucine and leucine and glutamine tended to increase upon CoCl_2_, while lysine and valine remained unchanged (Figure 3D). The acetate and formate levels in culture media, besides not being significant, presented a trend to increase and decrease, respectively (Figure 3E,F). The metabolic profile showed that endothelial cells exposed to CoCl_2_ presented decreased intracellular acetate levels, while formate and amino acid levels were not affected (Figure 3G–I).

### 3.3. Cysteine Does Not Affect the Sprouting of Aortic Rings and Tends to Shift the Metabolic Profile

**Figure 4:**
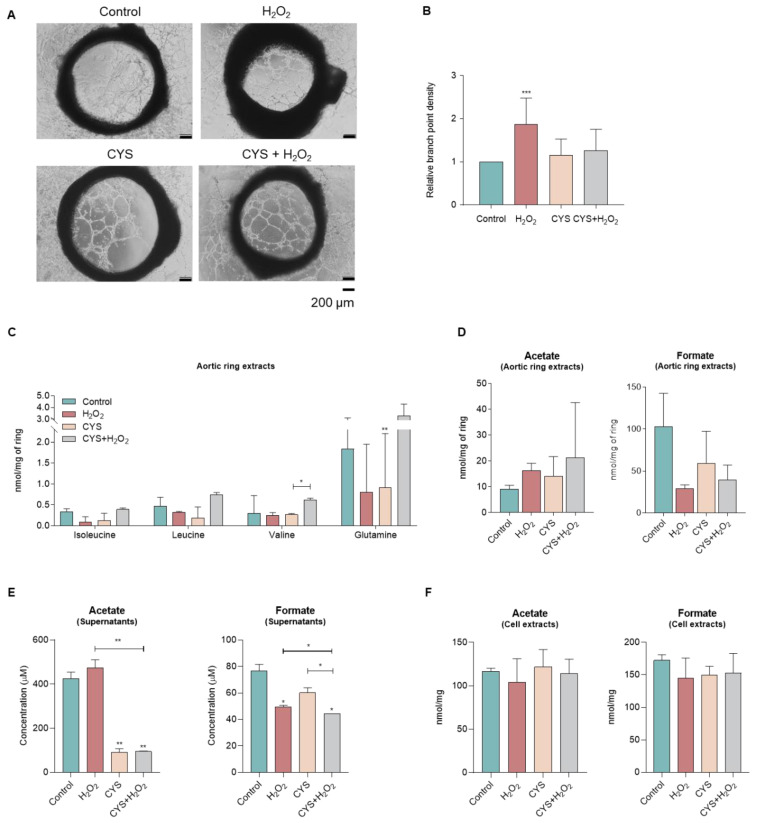

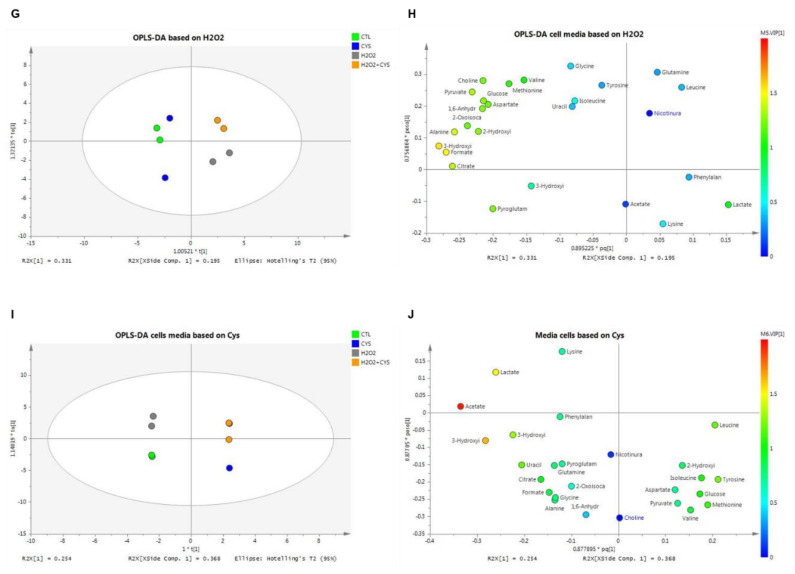
**Oxidative stress mimicked by H_2_O_2_ promotes aortic ring sprouting, while cysteine (CYS) reverses this effect.** Sections of rat aortic rings were plated in Matrigel and cultured in basal culture condition (control) and with hydrogen peroxide (15 μM, H_2_O_2_; ROS) in the presence or absence of cysteine (0.4 mM, CYS). Aortic rings’ endothelial cell sprouting was followed for 10 days. Cells isolated from aortic rings were cultured in the same conditions; then cell extracts were performed, and cell culture media (supernatants) were collected for nuclear magnetic resonance (NMR) spectroscopy analysis. (**A**) Representative images of rat aortic rings at day 10 (40×, scale: 200 μm). The rat aorta segments were exposed to H_2_O_2_ and CYS, alone and in combination. (**B**) Sprouting quantification: number of branch points *per* sprout area (area around and inside the ring where sprouting was visible), normalized to the control. (**C**) Metabolite concentration of BCAAs (isoleucine and leucine), valine, and glutamine of extracts of aortic rings exposed to H_2_O_2_ and/or CYS. (**D**) Acetate and formate levels in aortic ring extracts. (**E**) Acetate and formate levels in culture media of endothelial cells isolated from the sprouted aortic rings. (**F**) Acetate and formate levels of extracts from endothelial cells isolated from the sprouted aortic rings. Results are represented as mean ± SD. * *p* < 0.05, ** *p* < 0.01, *** *p* < 0.001; unpaired *t*-test (two-tailed)**.** Multivariate analysis of monocytes cultured under H_2_O_2_ and/or cysteine—PLS-DA model from supernatants, using (**G**) H_2_O_2_ and (**I**) cysteine as discriminant. Metabolites important for the separation of metabolic profiles upon (**H**) H_2_O_2_ and (**J**) cysteine exposure. The score values determined the position of the different samples on the OPLS-DA score plot, and the loading vectors the importance of the metabolites for the construction of the OPLS-DA model. The t1-X-score vector value of the first predictive component, the to1-orthogonal X-score vector of the first component, the pq1-predictive loading vector of the first component, and the poso1-orthogonal X-loading vector of the first component.

In order to understand the impact of ROS in the metabolic profile of endothelial cells organized in a real vessel structure, rat aortic rings were exposed to H_2_O_2_ and cysteine. H_2_O_2_ stimulated the sprouting of aortic rings, while cysteine rescued this effect (Figure 4A,B). Concerning the metabolic profile, a tendency to decrease BCAAs, valine, glutamine, and formate and to increase acetate upon ROS was observed, while cysteine reverted this profile at least in part (Figure 4C,D). The endothelial cells resulting from the sprouting of aortic rings were collected and exposed to the same experimental conditions and analyzed by NMR spectroscopy. The supernatant of these cultures showed a clear tendency to increase the levels of acetate upon ROS and a significant rescue of this effect by cysteine. Moreover, extracellular formate levels significantly decreased upon ROS exposure (Figure 4E). In the cell extracts, the levels of acetate and formate were maintained independently of the culture condition (Figure 4F). Interestingly, ROS exposure was enough to shift the metabolic profile of endothelial cells isolated from the sprouted aortic rings, leading to decreased levels of formate, alanine, and 3-hydroxybutyrate and to increased levels of lactate (Figure 4G,H). In agreement, cysteine exposure reverts the metabolic profile of these endothelial cells, by decreasing the levels of acetate, lactate, and 3-hydroxybutyrate (Figure 4I,J).

### 3.4. CoCl_2_-Mimicked Hypoxia Inhibits the Sprouting of Aortic Rings

**Figure 5:**
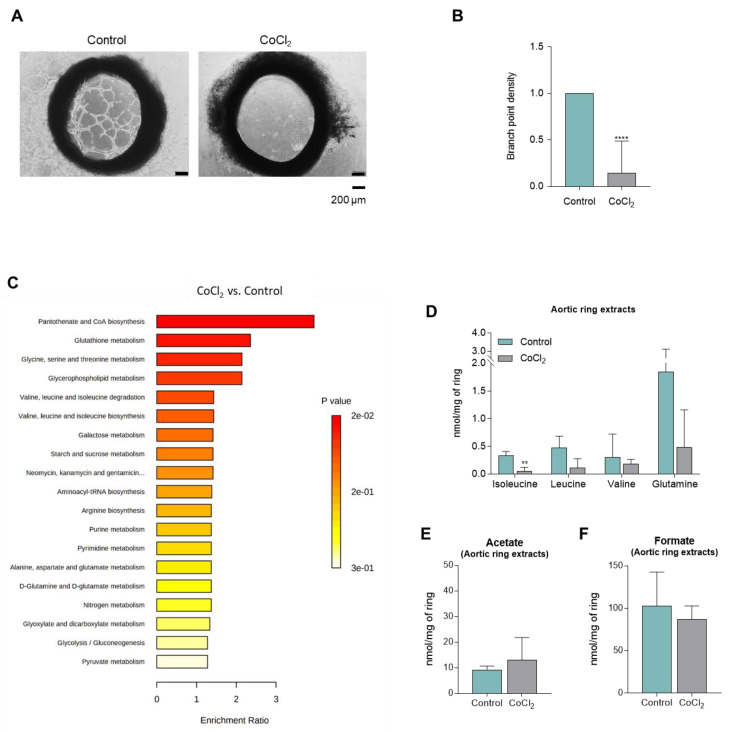
**Hypoxia inhibits the sprouting of aortic rings**. Sections of rat aortic rings were plated in Matrigel and cultured in basal culture condition (control) and in hypoxia-mimicked condition (200 μM CoCl_2_). Aortic rings’ endothelial cell sprouting was followed for 10 days. Aortic rings were collected, cell extracts were performed, and cell culture media (supernatants) were collected for nuclear magnetic resonance (NMR) spectroscopy analysis. (**A**) Representative images of aortic rings at day 10 (40×, scale: 200 μm). (**B**) Sprouting quantification: number of branch points *per* sprout area (areas around and inside the ring were sprouting and visible), normalized to the control. (**C**) Enrichment analysis of pathways overexpressed when aortic rings were exposed to CoCl_2_. (**D**) Metabolite concentration of BCAAs (isoleucine and leucine), valine, and glutamine of extracts of aortic rings exposed to CoCl_2_. (**E**) Acetate levels in aortic ring extracts. (**F**) Formate levels in aortic ring extracts. Results are represented as mean ± SD. ** *p* < 0.01, **** *p <* 0.0001; unpaired *t*-test (two-tailed).

The role of hypoxia was addressed by exposing aortic rings to CoCl_2_, and it was verified that CoCl_2_ inhibited the sprouting (Figure 5A). This inhibitory effect is clearly seen by the significant reduction of branch point density (Figure 5B). The exposure to CoCl_2_ induced a metabolic profile adjustment with an increase in glutathione, amino acids, glycids, purine, and pyrimidine metabolism (Figure 5C), together with a decrease in the levels of BCAAs, valine, and glutamine (Figure 5D). A trend for an increase in acetate and a decrease in formate levels was observed (Figure 5E,F).

### 3.5. Different Metabolic Profiles Correspond to the Activation of Endothelial Cell, the Sprouting of Aortic Ring Induced by ROS, and the Inhibition by Propranolol

**Figure 6:**
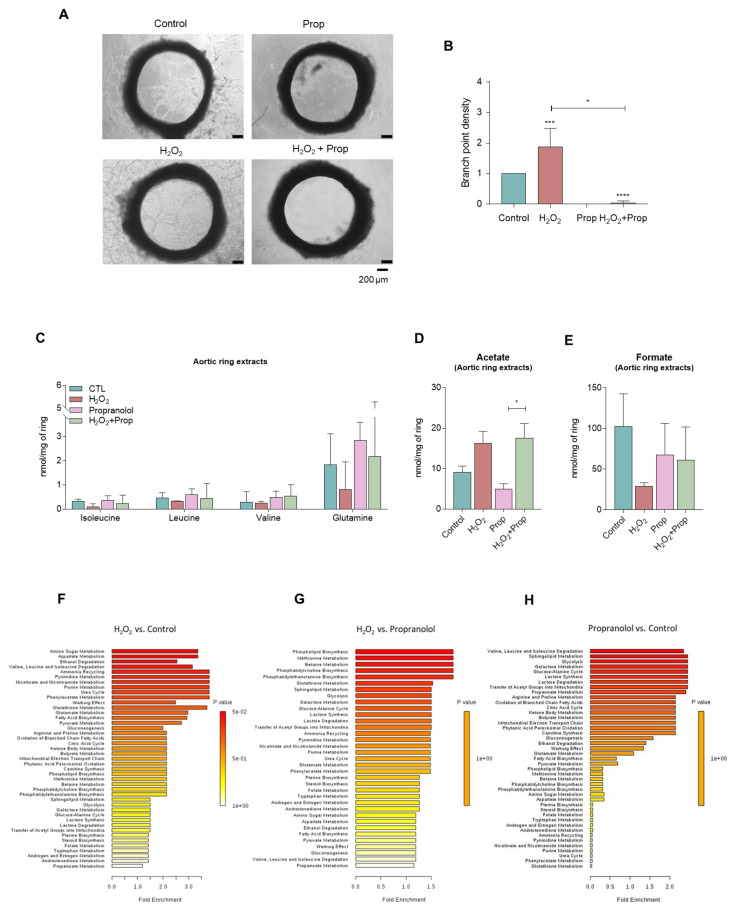
**Propranolol inhibits the sprouting of aortic rings**. Sections of rat aortic rings were plated in Matrigel and cultured in basal culture condition (control) and with hydrogen peroxide (15 μM, H_2_O_2_; ROS) in the presence or absence of propranolol (100 μM Prop). Aortic rings’ endothelial cell sprouting was followed during 10 days. Cells were collected, cell extracts were performed, and cell culture media (supernatants) were collected for nuclear magnetic resonance (NMR) spectroscopy analysis. (**A**) Representative images of aortic rings at day 10 (40×, scale: 200 μm). (**B**) Sprouting quantification: number of branch points *per* sprout area (areas around and inside the ring were sprouting and visible), normalized to the control. (**C**) Metabolite concentration of BCAAs (isoleucine and leucine), valine, and glutamine of extracts of aortic rings exposed to H_2_O_2_ and/or propranolol. (**D**) Acetate levels in aortic ring extracts. (**E**) Formate levels in aortic ring extracts. Results are represented as mean ± SD. * *p* < 0.05, *** *p* < 0.001, **** *p <* 0.0001; unpaired *t*-test (two-tailed)**.** Enrichment analysis of pathways overexpressed when aortic rings were exposed to (**F**) H_2_O_2_, (**G**), propranolol after H_2_O_2_, and (**H**) propranolol.

In order to find if the proangiogenic impact of ROS and the antiangiogenic effect of propranolol in endothelial cell functioning would correspond to different metabolic profiles, assays were performed with H_2_O_2_ and propranolol exposure. As seen in Figure 6A,B, H_2_O_2_ (ROS) stimulated the endothelial cell sprouting from aortic rings, and propranolol completely abrogated this effect even in the presence of H_2_O_2_ (Figure 6A,B).

The levels of BCAAs, valine, glutamine, and formate tended to decrease upon H_2_O_2_, and propranolol rescued this effect (Figure 6C,E). Regarding acetate, its levels were increased by ROS, and again, propranolol rescued this effect, when compared with control condition (Figure 6D). Overall, it was observed that aortic rings exposed to H_2_O_2_, and compared with control condition, presented increased valine, isoleucine, and leucine degradation, glutathione metabolism, pyruvate metabolism, nicotinate and nicotinamide metabolism, purine and pyrimidine metabolism, gluconeogenesis, and fatty acid biosynthesis and degradation and decreased folate metabolism, glycolysis, and glucose–alanine cycle (Figure 6F). Interestingly, this metabolic pattern in control and H_2_O_2_ conditions was rescued upon propranolol exposure (Figure 6G,H). Overall, the pathways activated by ROS, which accompany the sprouting of aortic ring, are concomitantly decreased upon exposure to propranolol and consequent sprouting inhibition. Importantly, this propranolol effect is seen even in basal control conditions.

## 4. Discussion

As we previously published, oxidative stress is a crucial stimulus for monocyte differentiation into endothelial cells, and it can be inhibited by cysteine [8], mainly due to its antioxidant role. Interestingly, the abrogation of monocyte-to-endothelial-cell differentiation upon cysteine exposure impaired BCAAs, valine, and glutamine catabolism induced by ROS exposure (Figure 2A), indicating that the antioxidant cysteine properties impair the metabolic readjustment occurring during ROS-induced differentiation of monocytes into endothelial cells. The same effect was also seen in the aortic ring sprouting assay (Figure 4A,B). Furthermore, the increased formate levels and the decreased acetate levels (Figure 2C), observed in monocytes under cysteine supplementation, may be related to a switch from differentiation into proliferation, as this acetate and formate pattern was previously linked to cell proliferation [30,31,32]. In fact, we speculate that cysteine stops the differentiation process of monocytes and activates their proliferative capacity, which is accompanied by a metabolic adjustment (Figure 1). Therefore, cysteine supplementation decreases one-carbon metabolism, glycine being deviated to produce ribulose-1,5-biphosphate and, afterwards, formate, instead of supplying the folate cycle that together with the methionine cycle contributes to the trans-sulfuration pathway for cysteine synthesis [30,31,32] (Figure 1). This is in agreement with the decreased folate metabolism observed in aortic rings, whose sprouting was inhibited by propranolol (Figure 6G,H). The decreased levels of 3-hydroxybutyrate also reinforced the impairment of one-carbon metabolism by cysteine (Figure 4J), since the ketone body degradation was recently indicated as responsible for one-carbon metabolism downregulation [33]. Additionally, the decreased acetate levels in monocytes are accentuated by cysteine, because in cells that are not in the differentiation process, and may be more proliferative, acetate supplies fatty acid synthesis [30,31,32] (Figure 1). Accordingly, in the aortic rings, propranolol inhibits endothelial cell sprouting, which is also sustained by proliferation, and this effect is concomitant with decreased lipid metabolism, including fatty acid synthesis (Figure 6F,G). However, more studies are needed to clarify the role of cysteine in cell proliferation and to disclose to what extent cysteine is used by endothelial cells to control oxidative stress or to supply biosynthesis and bioenergetics.

As we described recently, the ALDH inhibition by disulfiram activates monocyte-to-endothelial-cell differentiation [8], since ALDH is a stemness marker whose decreased expression and activity is correlated with a progressive differentiation of endothelial progenitor cells to endothelial cells [34,35]. Disulfiram did not present a great impact at the metabolic level, as no alteration was observed when tested in combination with any other condition (Figure 2F,G). Propranolol showed no effect on acetate levels (Figure 2F), which is in line with the previous observation that propranolol does not interfere with the monocyte-to-endothelial-cell differentiation [18].

**Figure 7:**
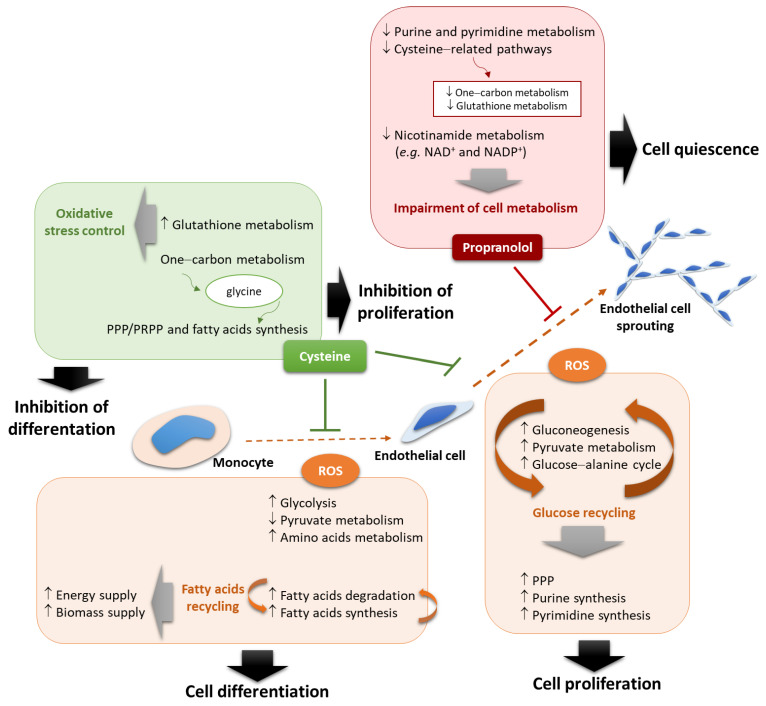
**Metabolic profiling is an indicative of cell biological processes.** ROS are a main stimulus for monocyte-to-endothelial-cell differentiation and endothelial cell proliferation. In this differentiation process, monocytes present increased glycolysis and amino acid metabolism with decreased pyruvate metabolism and fatty acid recycling. The ROS-dependent activation of proliferation is accompanied by a glucose-recycling metabolic adjustment, which will favor glucose-dependent pathways as the pentose phosphate pathway (PPP) and purine and pyrimidine syntheses. Cysteine inhibits monocyte-to-endothelial-cell differentiation and endothelial cell proliferation, with a concomitant upregulation of glutathione metabolism, and activates the glycine shuttle from one-carbon metabolism into the PPP/phosphoribosyl diphosphate pathway (PRPP) and fatty acid synthesis. Propranolol inhibits endothelial cell proliferation [18], and this accounts for a decrease in metabolic efforts, since a reversion of the metabolic pathways enhanced upon the stimulation of cell proliferation resembles a metabolic impairment consistent with cell quiescence. ↓—decrease; ↑—increase.

ROS are powerful stimuli for endothelial cell activation, from which increased proliferative capacity is an important component, always supported by metabolic adjustments (Figure 4 and Figure 7). Our results show that the metabolic adjustment required to sustain proliferation is evidenced by the fact that the Warburg effect is favored by ROS, and propranolol impairs this switch (Figure 6F,G), as well as hypoxia (Figure 5C). In the ROS condition, the activation of pyruvate metabolism and gluconeogenesis together with the decreased glycolysis and glucose–alanine cycle (Figure 6F,G) may indicate an internal loop of glucose recycling, feeding pivotal metabolic pathways, such as the pentose phosphate pathway (PPP) and purine and pyrimidine synthesis. All these dynamic flows are in agreement with putative metabolic symbioses acting between endothelial cells in different activation stages, since tip cells present a more glycolytic phenotype [36,37,38,39,40,41,42], whereas stalk cells present a profile more fitted to oxidative phosphorylation [36,43,44,45,46,47], being capable of using lactate (released by tip cells) and fatty acids as metabolic sources. Nonetheless, *in vitro* endothelial cells seem to be metabolically highly plastic [48].

The aortic ring sprouting assay is an *ex vivo* assay resembling partially the formation of new capillaries from pre-existing vessels. In this model, it was clear that the fatty acid oxidation and synthesis work simultaneously (Figure 6F,G), as it was already described in other studies [43,44,45,46,47,49,50,51]. However, here the lipid turnover relevance in angiogenesis is patented since ROS activate the sprouting of aortic rings, together with increased rates of fatty acid degradation and synthesis, these pathways being concomitantly decreased with the inhibition of sprouting by propranolol. Interestingly, glutathione metabolism increased upon ROS, which agrees with the role of cysteine and glutathione in the control of oxidative stress, allowing ferroptosis to occur at a nonlethal level and supporting endothelial cell activation [18].

Looking at the metabolic map, the results suggest that a cysteine-rich environment favoring a fast metabolism of cysteine supporting different metabolic pathways may favor cell proliferation through the glycine shuttle from the folate/methionine cycle to the PPP/phosphoribosyl diphosphate pathway (PRPP) and fatty acid synthesis (Figure 1 and Figure 7). Therefore, as we described previously, cysteine avoids monocyte differentiation into endothelial cells [8], and this process is certainly underlain to a metabolic rewiring, which is in agreement with the fact that monocytes upon the abrogation of ROS-dependent differentiation by cysteine present decreased acetate and increased formate levels (Figure 2D–G). Accordingly, the supernatant of cells sprouted from aortic rings presents the opposite dynamics for acetate and formate (Figure 4E), reinforcing that their measurement in a culture medium can be representative of endothelial cell activation or proliferation status. In the endothelial cells sprouted from aortic rings, acetate and formate were stressed again as important markers for proliferation, since H_2_O_2_ induces decreased formate levels, while exposure to cysteine prompts decreased levels of acetate (Figure 4G–J). Although aortic ring extracts do not present a significant result for acetate and formate dynamics, a tendency for increased levels of acetate and decreased levels of formate concomitant with sprouting inhibition was observed upon cysteine (Figure 4D) and hypoxia (Figure 5E,F). The nonsignificant results may be related to the fact that the aortic rings are not only composed of endothelial cells; the bulk of cells is connective tissue and smooth muscle cells, which can make it difficult to identify which are the metabolites produced by endothelial cells. Therefore, the supernatants are more representative of the metabolism of endothelial cells, which are the more proliferative and active components of the sprouting aortic rings. Moreover, propranolol inverted the levels of acetate and formate in aortic ring cells compared with the proactivating endothelial cell H_2_O_2_ condition (Figure 6D,E). Thus, it strengthens the fact that acetate can be used as a biomarker for *in vitro* monocyte differentiation into endothelial cells and *in vitro* and *ex vivo* endothelial cell proliferation and sprouting (Table 1), being useful to monitor the endothelial compartment behavior in tissue cultures.

This study takes one more step towards the understanding of the mechanism underlining the propranolol antiangiogenic effect, which is clearly translated by the metabolic profile and its interference with antioxidant mechanisms (Figure 7). Thus, propranolol reduces to the minimum some metabolic pathways that are linked to cell proliferation, such as purine and pyrimidine metabolism, and cysteine-related pathways, such as folate and methionine cycles and glutathione metabolism, and the synthesis of metabolic cofactors, such as the electron acceptor compounds (e.g., NAD^+^ and NADP^+^), which can be seen by the abrogation of nicotinamide metabolism (Figure 6G,H). The decreased glutathione metabolism (Figure 6G,H) reinforces the fact that the mechanism of action underlining the propranolol antiangiogenic effect is related to antioxidant control [18], maybe through cysteine degradation and hydrogen sulfide (H_2_S) production. It seems that, overall, propranolol induces a quiescent state in endothelial cells (Figure 7), agreeing with the reduced proliferation and migration rates concomitant with propranolol-related decreased ROS. We previously described this [18], and other studies indicated that low ROS are related to the induction of quiescence [52], resembling a metabolic remodeling, which includes the modulation of nicotinamide-derived compounds.

Metabolic profiling can be a powerful tool to follow the differentiation of endothelial progenitor cells, as monocytes, and to determine the formation of new vascular networks supporting *in vitro* tissue growth and viability in a perspective of regenerative medicine. This study is a step forward into a better knowledge of the metabolic adaptation needed for endothelial cell differentiation and activation/proliferation. Besides the metabolic shift affecting different metabolic pathways (Figure 7), the results suggested that acetate is a relevant biomarker for cellular alterations supporting the formation of vessel networks, and its concentration can be assessed by analyzing culture media without disturbing cellular/tissue and vascular structures. Importantly, the *in vitro* and *ex vivo* strategies used in this paper may contribute to improve the feasibility and success of human tissue transplantation, also diminishing the rejection events. The differentiation process of endothelial cells from monocytes allows for exploring the autologous transplantation of patient-derived cells constituting vascular networks. Furthermore, the sprouting of patient-derived vessels allows the isolation of endothelial cells to be used in *in vitro* vascular formation for further tissue culture, ensuring a proper tissue architecture and function while avoiding immunogenicity. Moreover, coculture, organoids, and tissue scaffolding assays will be important to validate biomarkers and to upgrade technical strategies towards the improvement of regenerative medicine.

## Figures and Tables

**Table 1:** Dynamics of acetate and formate in culture media (supernatants) and BCAAs (branched amino acids) in cell extracts of different *in vitro* endothelial cell models upon culture conditions related to inferred inhibition and activation of differentiation and proliferation processes.

		Supernatants		Cell Extracts	
	Culture Condition	Acetate	Formate	BCAA	Putative Biological Process
**Monocytes**	ROS (H_2_O_2_)	↑	↑	↓	↑ Differentiation
	Cysteine	↓	↑	↑	↓ Differentiation ↑ Proliferation
	Disulfiram	=	=	NT	↓ Differentiation
	Propranolol	=	=	NT	No effect
**HUVECs**	CoCl_2_	↑	↓	↑	↑ Proliferation
**ECAR**	ROS (H_2_O_2_)	↑	↓	↓	↑ Proliferation
	Cysteine	↓	↑	=	↓ Proliferation
	Propranolol	NT	NT	=	↓ Proliferation

HUVECs—human umbilical vein endothelial cells; ECAR—endothelial cells isolated from aortic rings, NT—not tested. ↓—decrease; ↑—increase.
